# Non-monotonic reorganization of brain networks with Alzheimer's disease progression

**DOI:** 10.3389/fnagi.2015.00111

**Published:** 2015-06-09

**Authors:** HyoungKyu Kim, Kwangsun Yoo, Duk L. Na, Sang Won Seo, Jaeseung Jeong, Yong Jeong

**Affiliations:** ^1^Department of Bio and Brain Engineering, Korea Advanced Institute of Science and TechnologyDaejeon, South Korea; ^2^Department of Neurology, Samsung Medical Center, Sungkyunkwan University School of MedicineSeoul, South Korea; ^3^Neuroscience Center, Samsung Medical CenterSeoul, South Korea

**Keywords:** Alzheimer's disease, complex network, resting state fMRI, functional connectivity, graph theory

## Abstract

**Background:** Identification of stage-specific changes in brain network of patients with Alzheimer's disease (AD) is critical for rationally designed therapeutics that delays the progression of the disease. However, pathological neural processes and their resulting changes in brain network topology with disease progression are not clearly known.

**Methods:** The current study was designed to investigate the alterations in network topology of resting state fMRI among patients in three different clinical dementia rating (CDR) groups (i.e., CDR = 0.5, 1, 2) and amnestic mild cognitive impairment (aMCI) and age-matched healthy subject groups. We constructed density networks from these 5 groups and analyzed their network properties using graph theoretical measures.

**Results:** The topological properties of AD brain networks differed in a non-monotonic, stage-specific manner. Interestingly, local and global efficiency and betweenness of the network were rather higher in the aMCI and AD (CDR 1) groups than those of prior stage groups. The number, location, and structure of rich-clubs changed dynamically as the disease progressed.

**Conclusions:** The alterations in network topology of the brain are quite dynamic with AD progression, and these dynamic changes in network patterns should be considered meticulously for efficient therapeutic interventions of AD.

## Introduction

Alzheimer's disease (AD) is the most common type of dementia, affecting about 5–10% of the population above the age of 65 (Sunderland et al., 2006). Clinical symptoms of AD are characterized by progressive amnesia, followed by a gradual decline in all cognitive functions, resulting in dementia (Sunderland et al., 2006). AD usually exhibits a typical clinical course reflecting the underlying progressing neuropathology (Bianchetti and Trabucchi, 2001; Storey et al., 2002). In the early stage, memory impairment is the prominent feature because the pathology initiates near the medial temporal cortex. In the moderate stage, language problems or visuospatial dysfunctions become conspicuous as the pathology propagates to the other temporal and parietal cortices (Förstl and Kurz, 1999; Bianchetti and Trabucchi, 2001). In the late stage of the illness, most cognitive functions are severely impaired, including frontal executive functions such as judgment, abstract, or logical reasoning, and planning (Braak and Braak, 1991; Fox et al., 2001). The mild cognitive impairment (MCI) is accompanied by mild memory deterioration but does not disrupt the activities of daily living. Although MCI is very heterogeneous and has multiple subtypes, it is considered to represent an intermediate stage between normal and dementia. MCI has been shown to be more likely to develop AD than cognitively normal (Boyle et al., 2006). Among MCI subtypes, Anmestic MCI (aMCI) is considered as a prodromal stage of AD, having a high-risk for progression to AD (Fischer et al., 2007). In addition, aMCI has been intensitvely investigated for early diagnosis of AD (Petersen, 2004).

Identification of clinical stages in Alzheimer's patients is crucial for the development of appropriate therapeutics that may delay the progression of the disease (Trojanowski et al., 2010). Several studies have attempted to determine the characteristics of each clinical stage of AD based on the distributions and patterns of the neuropathology (Braak and Braak, 1991), cognitive and behavioral performance (Folstein et al., 1975) and severity of clinical features such as the clinical dementia rating (CDR) (Hughes et al., 1982). For example, studies using structural MR imaging have shown that the regional patterns and rate of atrophy differ across AD stages (Scahill et al., 2002; Thompson et al., 2003). Atrophy in the medial temporal lobe commences prior to symptom onset, and is then followed by a reduction in gray matter in the limbic and other neocortices with relative sparing of primary sensory areas. These results are generally consistent with the tau accumulation and the corresponding clinical features (Scahill et al., 2002; Thompson et al., 2003).

In another aspect, there have been several studies supporting the hypothesis that AD is a “disconnection syndrome” (Delbeuck et al., 2003). According to this hypothesis, AD results from the disruption of neuronal connections due to synaptic loss and eventually neuronal death. This feature can be approached by adopting the concept of functional or structural connectivity. As the severity of the disease increases, the functional connectivity among brain regions is assumed to be gradually reduced. Indeed, prior neuroimaging studies have shown that AD patients show disrupted white matter integrity and functional connectivity among distant brain regions (Celone et al., 2006; Zhang et al., 2007). However, how different degrees of disruptions of connectivity in AD across clinical stages influence global information processing of the brain is not clearly understood yet (Wang et al., 2006; Zhou et al., 2008; Bai et al., 2011).

To address this question, the current study employed complex network analysis methods and investigated brain network properties from resting state fMRI in AD, aMCI, and healthy subjects (HS) (Strogatz, 2001; Bullmore and Sporns, 2009). Complex network analysis has shown that the brain has non-random network properties including small world and scale free features and exhibits hierarchical organization with modularity (Achard et al., 2006; Hagmann et al., 2008). Several fMRI and MEG studies have reported that AD and aMCI patients have reduced small-worldness characterized by a longer characteristic path length than that of HS (Stam et al., 2007, 2009; Kendi et al., 2008; Lo et al., 2010). However, we should note that some network properties in AD are not consistent across studies (for reviews, Xie and He, 2011; Tijms et al., 2013), for example, different studies have found increases (Kendi et al., 2008), decreases (Stam et al., 2009), or no significant changes in the clustering coefficient of the brain network in AD patients (Stam et al., 2007; Lo et al., 2010; Sanz-Arigita et al., 2010). Moreover, some studies have reported conflicting results regarding the characteristic path length; one study showed a decrease in the characteristic path length in AD brains (Sanz-Arigita et al., 2010), whereas another study reported no change (Supekar et al., 2008). This discrepancy regarding the change in the network topology of the AD brain might result from the heterogeneity of patient populations at different clinical stages of AD or the different strategy of generating network in the previous studies. To overcome this, we attempted to use the uniformly measured data sets and to raise the stability of network topology during the constructing networks. The parameters of realignment and segmentation of MR images were chosen based on the previous neuroimaging studies using SPM (Della-Maggiore et al., 2002; Maldjian et al., 2003). The density threshold values were also determined as to show the clear features of the brain network as the density value changes in a wide range. The threshold values for the same density network are in Supplementary Table 1.

To the best of our knowledge, there have been no previous studies using graph theoretical measures to investigate the consecutive changes in brain network topology with AD progression. Therefore, the current study investigated and compared topological properties of the brain networks in AD patients in three different CDR stages, patients with aMCI, and age-matched HS.

## Materials and methods

### Participants

A total of 278 subjects (112 AD, 87 aMCI, and 79 HS) were recruited consecutively at the memory disorder clinic in the Department of Neurology at Samsung Medical Center in Seoul, South Korea between March 2008 and February 2009. Each participant underwent MR scans, clinical interviews, neurological examinations, and comprehensive neuropsychological assessments. Patients with aMCI met the criteria proposed by Petersen et al. (1999). We diagnosed aMCI based on criteria of −1.5 to −1 SD of SVLT score. Patients with AD fulfilled the criteria for probable AD proposed by the National Institute of Neurological and Communicative Disorders and Stroke and the AD and Related Disorders Associations (NINCDS-ADRDA) (McKhann et al., 1984). AD patients were subdivided into three groups according to their CDR (Morris, 1993), 36 with a CDR of 0.5, 55 with a CDR of 1, and 22 with a CDR of 2. The HS group was comprised of 79 subjects with no history of cognitive impairment or neurological or psychiatric illness, and the subjects exhibited normal performance during neuropsychological testing. During various phases, 126 subjects were excluded from the study, and data from 152 subjects was included in the analysis.

While reviewing their neuropsychological tests, we excluded 31 subjects (5 NL, 6 aMCI, 3 AD patients with a CDR score of 0.5, 10 AD patients with a CDR score of 1, and 7 AD patients with a CDR score of 2 whose clinical information was incomplete on at least one neuropsychological item. We then constructed an individual brain network according to the method described in the following Section Construction of a Brain Network Using Resting State fMRI, but with a threshold of 0.8. We excluded outliers with network degrees below [the first quartile −1.5 times the interquartile range] or above [the third quartile +1.5 times the interquartile range]. The networks with small degrees during the measurement were considered to be noisy data, and the networks with large degrees could be affected by artifacts. The number of outliers excluded in this step was 82. We then again checked group-wise average ages. To match the average age among groups, we additionally excluded 13 subjects in the HS group. Every participant or their caregivers in this study provided written informed consent. This study was approved by the Institutional Review Board of Samsung Medical Center, Seoul, South Korea.

### Neuropsychological assessments

Each participant underwent neuropsychological testing using the Seoul Neuropsychological Screening Battery (SNSB), a standardized neuropsychological battery that includes validated tests for a variety of cognitive functions such as attention, language, visuospatial function, verbal, and visual memory, frontal executive function, and CDR (Kang and Na, 2003). Among these evaluations, scorable tests included the Digit Span Backward, the Korean version of the Boston Naming Test (K-BNT) (Kim and Na, 1999), the Rey-Osterrieth Complex Figure Test (RCFT) (Lezak, 1983), the Seoul Verbal Learning Test (SVLT; three learning-free recall trials of 12 words, a 20 min delayed recall trial for these 12 items, and a recognition test), motor tests (Contrasting program, a Go/NoGo test), the phonemic and semantic Controlled Oral Word Association Test (COWAT), and the Stroop Test (Color reading of 112 items during 2 min).

### Acquisition and preprocessing of MRI

MR images were acquired using a 3 Tesla MR scanner (Philips Intera Achieva, Philips Healthcare, The Netherlands). T1-weighted anatomical MR images (TR = 9.9 ms; TE = 4.6 ms; flip angle = 8°; FOV [FH, AP, RL] = 240 × 240 × 180 mm^2^; matrix = 480 × 480; 360 slices [sagittal]; voxel size = 0.5 × 0.5 × 0.5 mm^3^) and T2^*^-weighted MR images (resting state fMRI) were obtained using a gradient echo planar imaging pulse sequence (TR = 3000 ms; TE = 35 ms; flip angle = 90°; FOV [RL, AP, FH] = 220 × 220 × 140 mm^2^; matrix = 128 × 128; 35 slices [transverse]; voxel size [RL, AP, FH] = 1.72 × 1.72 × 4 mm^3^).

Pre-processing steps for resting state fMRI included slice-timing correction, motion correction, co-registration, segmentation, spatial normalization into Montreal Neurological Institute (MNI) space, and smoothing as described previously (Yoo et al., 2013). Pre-processing was performed using Statistical Parametric Mapping software 8.0 (SPM, http://www.fil.ion.ucl.ac.uk/spm/) in MATLAB R2011a (7.12).

### Construction of a brain network using resting state fMRI

We selected 90 brain regions as nodes to construct a brain network using an Automated Anatomical Labeling (AAL) parcellation scheme (Tzourio-Mazoyer et al., 2002). To determine the functional connectivity (i.e., the edges) between the nodes, we calculated mutual information for each pair of 90 fMRI time series extracted from each node. We then constructed a network with the same density for each individual and containing the same number of edges in every individual graph to facilitate comparison of network properties. This allows the comparison between groups with a controlled number of nodes and edges, because network properties with the same threshold change dramatically with their number of degrees.

$$\text{Mutual information}=\sum_{y}\sum_{x}p(x,y)log\left(\frac{p(x,y)}{p\left(x\right)p\left(y\right)}\right)$$

where *p(x,y)* is the joint probability distribution function of X and Y, *p(x)* and *p(y)* are the marginal probability distribution functions of X and Y, and X, Y are the serial values of fMRI from selected two regions in this study.

The various density values were tested to construct brain networks. Among them, we finally selected the lowest fixed density that provided a sufficiently sparse network with a lower bound of density, minimizing the number of isolated nodes. The threshold of each network was applied separately for fixed density, 7%. The density equals the number of edges divided by possible connections in the network,

$$\text{Density}=\frac{2E}{N(N-1)}$$

wherein the number of nodes *N* and edges *E*. This allows the controlled comparison of network structure and properties among different groups.

### Graph theoretical analysis of the brain network

After constructing an individual brain network for each subject, graph theoretical analysis was performed to obtain the topological information of the network. We then compared the whole brain network properties among the five groups (HS, aMCI, CDR 0.5, CDR 1, and CDR 2, ANOVA, and *post-hoc*). We compared seven network parameters, including the characteristic path length, clustering coefficient, global efficiency, local efficiency, betweenness centrality, assortativity, and modularity. The characteristic path length is the average of the minimum number of edges that have to be passed through between nodes. The clustering coefficient of a node is the rate of existing edges between the nearest neighbors vs. possible connections. Global and local efficiency indicate the information transfer between nodes. Global efficiency is the average of the inverse of the shortest path lengths of individual nodes. The local efficiency of an individual node is the inverse of the shortest path length connecting all neighbors of that node. The betweenness centrality of a single node is the number of shortest paths between nodes that must pass through the selected node dividing by the number of all paths to normalize. Assortativity is the correlation between the degrees of connected nodes. A positive assortativity indicates that high-degree nodes tend to connect to each other. The modularity is the fraction of the edges that fall within the given groups minus the expected fraction if edges were distributed at random. This analysis was performed using the Brain connectivity toolbox (Rubinov and Sporns, 2010). We also investigated the network properties of each brain lobe (frontal, parietal, occipital, and temporal lobes and the subcortical area). To do this, we first calculated nodal values for each of the 90 AAL ROIs and then took the average of these values within each lobe or area. We compared these lobar or areal properties across the five groups with AD progression (ANOVA and *post-hoc*). In this lobar analysis, we supposed that tests for each lobe are independent, hence no correction was applied.

### Correlation analysis between network topology and clinical information

We tested whether network properties were correlated with clinical measures by two methods. First, we examined whether a significant correlation existed between network properties and clinical information within each of the five groups. Second, we performed the same correlation analysis including all subjects regardless of groups. We calculated the Pearson's correlation coefficient between the network properties and clinical measures and determined the significance of correlation based on the *p*-value (*p* < 0.05). For the correlation analysis, uncorrected *p*-value was used.

### Rich-club organization

The rich-club in a complex network is a group of hubs having dense connections among themselves. The rich-club organization provides important information on the higher-order hierarchical backbone structure of networks (Colizza et al., 2006; McAuley et al., 2007). The rich-club phenomenon in networks is designed to measure when the hubs of a network tend to be more densely connected among themselves than nodes of a lower degree (Colizza et al., 2006). Networks having a relatively high rich-club coefficient show the rich-club effect and have many links between high degree nodes. The rich-club coefficient of a network is can be a measurement of the robustness. The networks are usually resilient with high rich-club coefficient, because the densely connected hubs can maintain the network structure easily. The rich-club coefficients of networks were also calculated in each group. We computed the rich-club coefficients Φ(*k*) of the networks over a range of degrees (*k*). For a degree (*k*), the edges and nodes with a smaller degree than *k* were removed from the network. In the remaining network, the rich-club coefficient Φ(*k*) is the ratio of the current edges and possible number of edges among remaining nodes,

$$\Phi \left(k\right)=\frac{2E_{>k}}{N_{>k}\left(N_{>k}-1\right)}$$

where *N* is the number of remaining nodes and *E* is the number of current edges. The rich-club coefficient Φ(*k*) can be normalized with a set of random networks of the same size and similar degree distribution. A normalized rich-club coefficient Φ_*norm*_ of > 1 can be described as a rich-club organization in a network (Colizza et al., 2006). We calculated the rich-club curve comparing the rich-club coefficient between subject groups and 1000 random networks by rewiring edges with a similar degree distribution for each level of *k*,

$$\Phi_{norm}\left(k\right)=\frac{\Phi \left(k\right)}{\Phi_{random}\left(k\right)}$$

We also determined whether the permutation test from the 1000 random networks was statistically significant. We showed that Φ(k) was significantly greater than the distribution of Φ_*random*_(*k*), with a *p* < 0.05.

### Statistical test

For the statistical tests, we used One-Way ANOVA for group difference of each property, and the Tukey's honest significant difference (HSD) test for *post-hoc* test in every comparison. The Tukey's HSD was optimal for One-Way ANOVA and similar procedures with equal sizes originally. As you already know, it has been confirmed to be conservative for One-Way ANOVA with the different sample sizes as well.

## Results

The demographics and neuropsychological results of subjects in the current study are listed in Table 1. There were significant differences among groups, with the higher CDR group showing poorer performance in every cognitive domain.

**Table 1:** **Demographics and clinical information**.

	**HC**	**aMCI**	**AD CDR 0.5**	**AD CDR 1**	**AD CDR 2**
Subjects	31 (26F)	50 (28F)	25 (15F)	36 (20F)	10 (6F)
Age	67.6 (±6.3)	70.4 (±7.6)	70.0 (±8.4)	72.6 (±7.7)	70.9 (±6.9)
Digit span backward	4.3 (±1.5)	3.5 (±1.0)	2.9 (±1.3)^aaa^	2.8 (±1.3)^aaa^	2.9 (±1.1)^a^
K-BNT	48.5 (±7.5)	40.6 (±9.3)^aa^	35.0 (±12.5)^aaa^	36.5 (±10.1)^aaa^	30.6 (±11.7)^aaab^
RCFT copy	32.2 (±5.0)	29.9 (±5.2)	23.8 (±11.4)^aab^	23.4 (±9.9)^aaabb^	21.4 (±10.9)^aab^
SVLT delayed	7.4 (±2.0)	2.5 (±2.4)^aaa^	1.0 (±1.9)^aaab^	0.6 (±1.1)^aaabbb^	0.4 (±1.3)^aaab^
RCFT delayed	15.0 (±3.9)	7.9 (±5.2)^aaa^	2.6 (±2.8)^aaabbb^	1.7 (±3.0)^aaabbb^	1.1 (±1.9)^aaabbb^
Contrasting program	19.9 (±0.2)	19.7 (±1.4)	18.4 (±4.5)	15.4 (±7.2)^aaabbb^	11.4 (±8.6)^aaabbbccc^
Go/NoGo test	19.6 (±1.1)	18.7 (±3.1)	16.1 (±5.3)	12.0 (±6.7)^aaabbbcc^	8.2 (±6.7)^aaabbbccc^
Stroop test color reading	87.7 (±23.3)	73.8 (±23.9)	48.8 (±29.5)^aaabbb^	39.5 (±25.1)^aaabbb^	20.6 (±16.0)^aaabbbc^
MMSE	28.6 (±1.9)	26.5 (±2.2)^a^	22.4 (±3.9)^aaabbb^	19.6 (±4.0)^aaabbbcc^	17.0 (±3.4)^aaabbbccc^
CDR sum of boxes (SNSB)	0.7 (±0.5)	1.3 (±0.8)	3.1 (±1.3)^aaabbb^	5.2 (±1.4)^aaabbbccc^	10.8 (±1.9)^aaabbbcccddd^
COWAT semantic	32.4 (±7.5)	24.4 (±7.5)^aaa^	21.4 (±6.9)^aaa^	18.2 (±6.9)^aaabbb^	12.9 (±5.3)^aaabbbc^
COWAT phonemic	28.1 (±10.8)	18.4 (±9.7)^aaa^	17.6 (±11.1)^aaa^	13.5 (±8.3)^aaa^	11.8 (±8.8)^aaa^

*ANOVA and post-hoc analysis was performed*.

*Significantly different when compared to normal (a: p < 0.05, aa: p < 0.01, aaa: p < 0.001)*.

*Compared to aMCI (b: p < 0.05, bb: p < 0.01, bbb: p < 0.001)*.

*Compared to AD CDR 0.5 (c: p < 0.05, cc: p < 0.01, ccc: p < 0.001)*.

*Compared to AD CDR 1 (ddd: p < 0.001)*.

**Figure 1:**
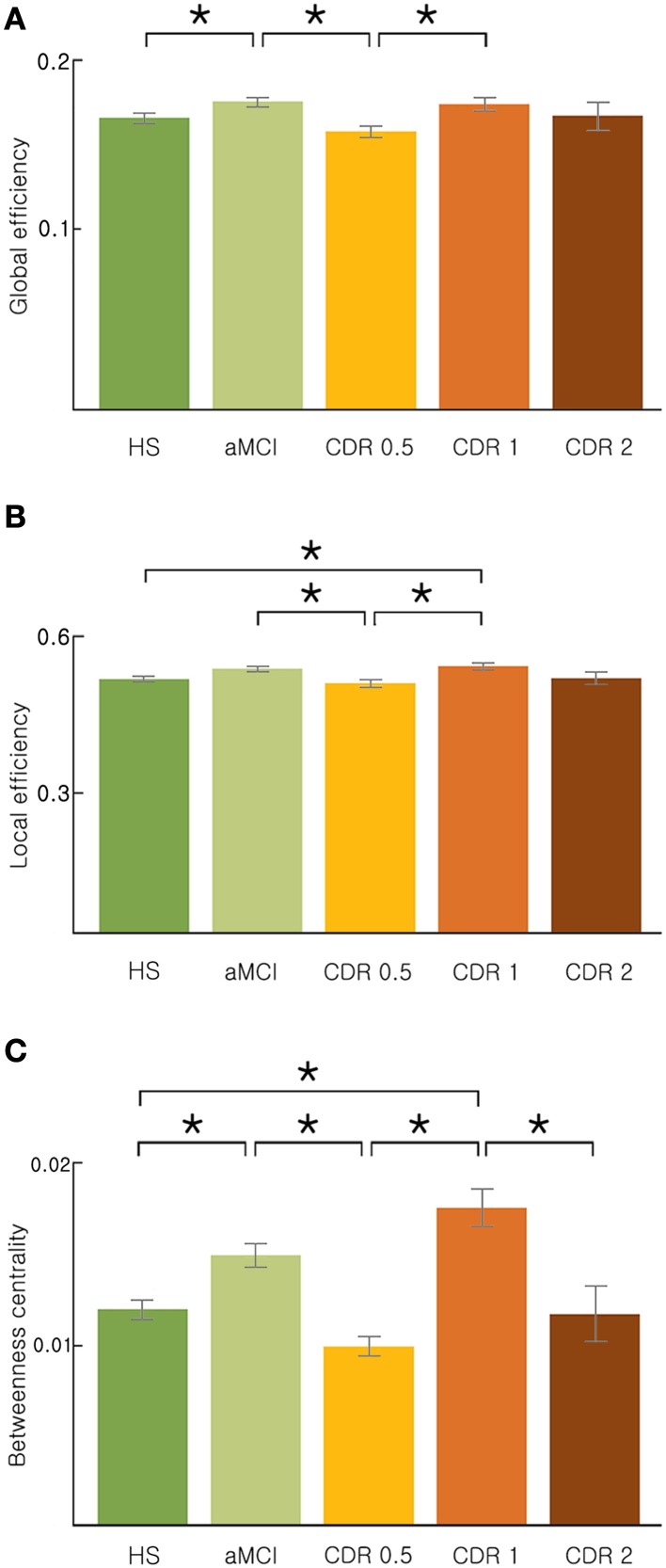
**Topological properties of the AD brain networks with the disease progression. (A)** Global efficiency, **(B)** local efficiency, and **(C)** betweenness centrality changed stage-specifically in a non-monotonic manner. Significance is represented by an asterisk (*p* < 0.05, ANOVA and *post-hoc*).

We investigated changes in the topology of brain networks spanning from HS, to subjects with aMCI, to subjects in the AD spectrum. Figure 1 shows the reorganization of the network from the HS to AD patients with a CDR of 2 (ANOVA and *post-hoc*) with respect to three parameters: global efficiency, local efficiency, and betweenness centrality. We found a non-monotonic change in the brain network as AD progressed; these 3 network properties are higher in aMCI and CDR 1 groups than other three groups. In common, these three network properties were significantly lower in the CDR 0.5 group compared with the aMCI group or the CDR 1 group (*p* < 0.05, uncorrected). In addition, we estimated the characteristic path length, clustering coefficient, modularity, and assortativity and found similar fluctuating patterns with AD progression (see Supplementary Figure 1).

We then determined whether the topology of the brain network correlated with the performance of particular cognitive functions. We performed a correlation analysis between network measures and neuropsychological test scores for each group separately (Table 2 and Supplementary Table 2). We found significant correlations between network properties and neuropsychological test scores primarily in the aMCI group (*p* < 0.05, uncorrected). The aMCI group showed a negative correlation between the scores of the Digit Span Backward, Naming K-BNT, RCFT copy, Go/NoGo, and COWAT Semantic tests and network properties. The HS showed significant correlations between the Stroop Test scores and network measures (global efficiency, betweenness centrality, and characteristic path length). In addition, the CDR 0.5 group showed significant positive correlations between the COWAT Phonemic score and betweenness centrality and characteristic path length. In contrast, no significant correlations were found between neuropsychological test performances and network measures in the CDR 1 and CDR 2 groups. All significant results were negative correlations (except those found in the CDR 0.5 group) and are shown in Table 2 and Supplementary Table 2. In addition, we performed the same analysis for all subjects and found a significant negative correlation of the COWAT semantic score with network topologies (betweenness centrality, characteristic path length, and clustering coefficient, *p* < 0.05, uncorrected, Supplementary Table 3).

**Table 2:** **Correlation between network properties and clinical information within each group (*p* < 0.05, uncorrected, *p*/*R*^2^/*r*)**.

		**Global efficiency**	**Local efficiency**	**Betweenness centrality**
HS	Stroop test color-reading correct	0.035/0.145/−0.381	–	0.025/0.162/−0.402
aMCI	Digit span backward	–	–	0.028/0.096/−0.310
	Naming K-BNT	–	0.021/0.106/−0.325	0.022/0.104/−0.323
	RCFT copy	–	0.028/0.096/−0.310	–
	Go/NoGo	–	0.010/0.131/−0.361	0.025/0.100/−0.317
	COWAT semantic	0.029/0.096/−0.310	0.010/0.131/−0.361	0.010/0.132/−0.363
AD CDR 0.5	COWAT phonemic	–	–	0.031/0.187/0.433

*HS, healthy subject; aMCI, amnestic mild cognitive impairment; AD, Alzheimer's disease; CDR, clinical dementia rating*.

*R^2^: goodness of fit by coefficient of determination; r: Person's correlation coefficient*.

**Figure 2:**
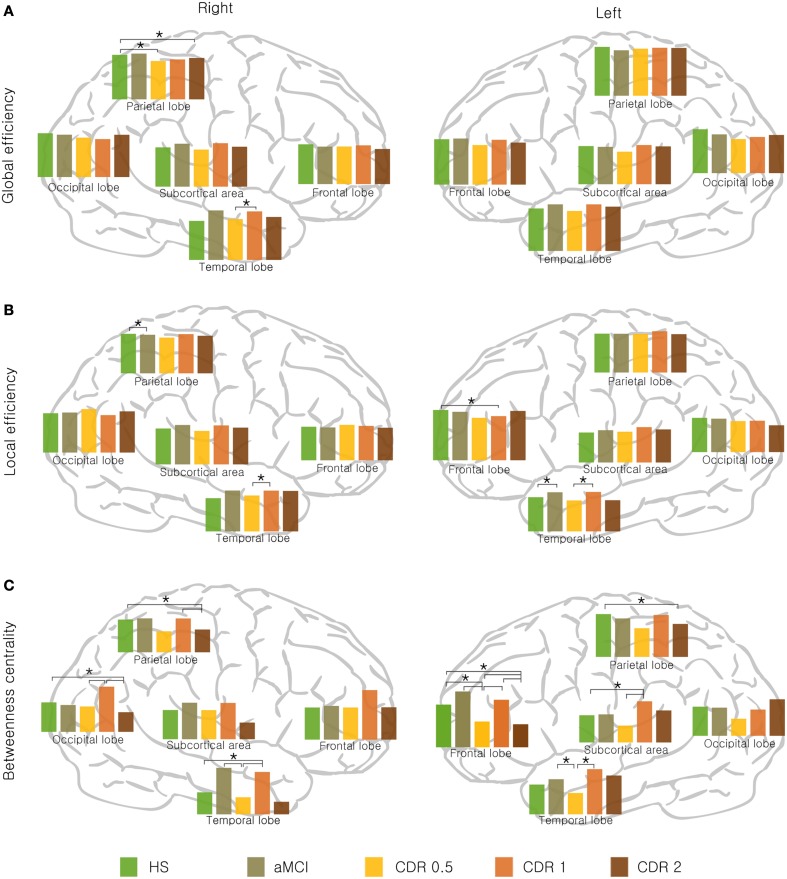
**Lobar network properties with AD progression. (A)** Global efficiency, **(B)** local efficiency, and **(C)** betweenness centrality changed stage-specifically in a non-monotonic manner. Significance is represented by an asterisk (*p* < 0.05, ANOVA and *post-hoc*).

Next, we examined whether the changes in the network properties of each lobe were reflected in whole-brain topology as AD progresses. For each of the three network measures, global, and local efficiency and betweenness centrality, the bilateral temporal lobe and right subcortex showed a non-monotonic reorganization with AD progression (Figure 2). Particularly, the left frontal lobe exhibited this reorganization pattern for global efficiency and betweenness centrality, whereas the left subcortex exhibited this reorganization with respect to local efficiency and betweenness (Figure 2). In addition, the right frontal and parietal lobes displayed similar non-monotonic reorganization only for betweenness centrality.

We determined if there were significant changes in lobar network properties among AD groups. For global efficiency, the right parietal lobe showed significantly lower efficiency in the CDR 0.5 and CDR 2 groups compared to the HS group (*p* < 0.05), and the right temporal lobe showed an higher in the CDR 1 group compared with the CDR 0.5 group (*p* < 0.05). For local efficiency, the right parietal lobe showed lower efficiency in the aMCI compared to the HS group (*p* < 0.05), and the right temporal lobe was higher in the CDR 1 group compared to its preceding group, CDR 0.5 (*p* < 0.05). In left hemisphere, the temporal lobe was higher in the aMCI and CDR 1 groups compared to the HS and CDR 0.5 groups, respectively (*p* < 0.05). All significant results are shown in Figure 2. Other network properties, i.e., the characteristic path length, clustering coefficient, modularity, and assortativity, also exhibited a similar pattern of change with AD progression (see Supplementary Figure 2).

**Figure 3:**
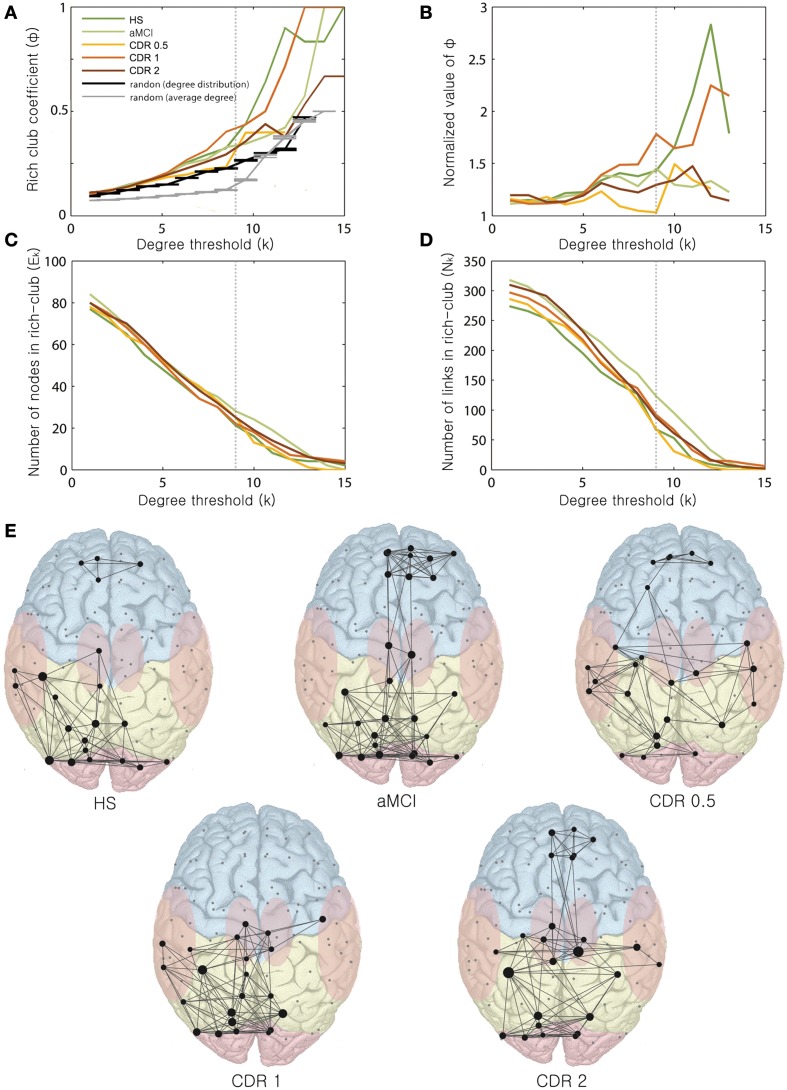
**Changes in rich-club organization as a function of k**. Changes of the **(A)** rich-club coefficient, **(B)** normalized rich-club coefficient, **(C)** number of nodes within the rich-club, and **(D)** number of links within the rich-club are displayed with varying *k*-values ranging from 0 to 15. **(E)** Rich-club organization with AD progression. Representative networks for each stage are shown for *k* = 9.

Lastly, we examined the rich-club organization of the brain networks with AD progression. Figures 3A–D) shows the rich-club coefficient, normalized rich-club coefficient, number of nodes, and number of edges as a function of degree threshold k from 1 to 15 for AD patients in different CDR stages and HS. For *k*-values larger than 9, the largest normalized rich-club coefficient was observed in the HS group, followed by the CDR 1 group. The remaining groups showed similar normalized rich-club coefficients within a range of 1–1.5. For *k*-values equal to 9, the aMCI and CDR 1 groups exhibited higher original and normalized rich-club coefficients and a greater number of links within the rich-club organization relative to the other AD stages.

We observed varying distribution patterns of rich-club nodes for the degree threshold *k* = 9 across AD stages (Figure 3E). For a *k* of 9, the numbers of nodes of the rich-clubs were 21, 28, 25, 22, and 25, and the numbers of edges were 68, 124, 70, 93, and 88 from the HS to the CDR 2 group. The original rich-club coefficients for *k* = 9 were 0.32, 0.33, 0.23, 0.4, and 0.29, and the normalized rich-club coefficients were 1.41, 1.42, 1.05, 1.76, and 1.30 from the HS to the CDR 2 group. The rich-club coefficients of the original and normalized values and the number of edges within the rich-club varied non-monotonically, similar to the change of the whole brain network properties as AD progresses. Within the rich-club core of the brain, frontal regions were not connected with posterior regions in the HS group. In contrast, in the aMCI group, the rich-club consisted of an increased number of frontal regions, and those were well-connected with the other posterior (parietal and occipital) regions. Interestingly, these frontal regions and their connectivity gradually decreased and disappeared in the CDR 1 group before increasing again in the CDR 2 group.

## Discussion

We investigated changes in network properties from HS to subjects with prodromal and intermediate stages of AD by comparing topological measures of the brain network and determining their relationship with behavioral and clinical test scores. The current study first examined the whole process of alterations in brain networks from the time before AD onset to a severe AD stage. To properly examine and compare the topological reorganization of the brain network, it was necessary to construct networks with similar size. In the current study, we constructed a brain network with the same density (sparsity), avoiding the use of a specific threshold value. Because the density network contains the same number of edges, we were able to compare brain networks of the same size among groups.

We demonstrated the ongoing reorganization process of the brain network with AD progression. However, we did not observe a correlation between any network measure and clinical deterioration, e.g., CDR. Unexpectedly, this reorganization occurred stage-specifically in a non-monotonic manner (Figure 1 and Supplementary Figure 1). It has been proposed that the progression of AD follows a sigmoidal curve (Jack and Holtzman, 2013). However, the smooth progressive change in each parameter is rather presumptive and mainly based on interpolation or extrapolation of limited evidence. Given the five stages of AD progression, we revealed that network topological properties, including network efficiency and betweenness centrality, were higher in the aMCI and CDR 1 groups compared to other AD groups or HS. First, the brain network in the aMCI group exhibited significantly higher efficiency compared to that of the previous stage, the HS group. Higher network efficiency and betweenness centrality would result from the presence of additional hub regions. In addition, the results from the correlation analysis between neuropsychological test scores and network properties support a stage-specific non-monotonicity. We found that correlations between neuropsychological scores and network properties were distinguishable among each group of AD progression (Table 2, and Supplementary Tables 2, 3). The speculation for this finding is described in Supplementary Material.

It is interesting and unusual that an advanced disease stage has higher network efficiency than a previous stage. We speculate that this finding is in line with previous studies reporting hyperactivation in the hippocampus and other memory-related areas during cognitive and memory-related tasks in MCI patients (Dickerson et al., 2005; Hämäläinen et al., 2007) compared to HS and AD patients. In addition to the task-induced activation, increased resting state connectivity in aMCI patients compared to HS has also been reported (Sohn et al., 2014). Another study demonstrated that MCI patients exhibiting faster cognitive decline have greater hippocampal activation (Miller et al., 2008). Given that the resting state connectivity and the brain activation show a positive correlation with each other (Mennes et al., 2011), our result of increased brain network efficiency in aMCI patients is consistent with the aforementioned studies. The reorganization process of the rich-club core with AD progression is speculated in detail with other possibilities and scenarios of non-monotonic changes in AD brain networks in Supplementary Material.

We should be cautious in interpreting the results, because there are several limitations and ambiguous outcomes from these analyses. Moreover, we should note that these results appear to contrast with those of other previous studies. Based on this discrepancy, it is likely uncertain whether the non-monotonic changes in network parameters are generated by disease progression or not. We also mention that the mechanisms underlying the connections between network parameters and disease progression are not clear, because the parameters tended to be non-monotonic. Another recent study showed increasing path length and decreasing small-worldness of the density network with AD progression (Sun et al., 2014). This can be seen as opposite contrasting result with our study, although that study used different group stratification and different measurements of network construction compared with this study. We chose mutual information as a measure of functional connectivity, whereas the most common measure is the Pearson's correlation coefficient. The Pearson's correlation provides information about the linear relationship between regions but does not detect non-linear interactions among regions. Therefore, as an alternative approach that accounts for non-linear interactions, we used mutual information to construct the information transmission network of the brain. However, the path length used in the current study showed a mildly significant increase with the disease progression, whereas Sun et al. showed some regional betweenness centrality changes in non-monotonic values with disease progression. This discrepancy in the results between the studies using similar data and methods suggest that other elements may influence these network properties and indicate our limited understanding of the innate causal relationship between network parameters and AD. Based on these potential explanations for the observed non-monotonic network parameter changes in the current study, the possibility that our conclusions are incorrect or produced from other influences may not be excluded.

Rich-club organization also plays an unclear role in these results. Differences in rich-club network structure were observed not only between the groups exhibiting significantly different network parameters but also between groups exhibiting similar values. This makes our results difficult to interpret because the innate cause of rich-club structure changes remains still unclear. We should admit that the *post-hoc* analysis did not produce significant results in the analysis of lobar parameters. Because of the insufficient number of subjects in some groups, it is likely assumed that the lobar parameters are independent of each other. The *post-hoc* tests were calculated only for group dependency. This limitation suggests that we should interpret our major finding of the non-monotonic changes in the lobar parameters with extreme caution. In addition, we noted that HS, aMCI and AD severity in each CDR category were continuous variables in this analysis; however, since this study was a cross-sectional study, a longitudinal analysis is required to investigate the changes in each patient over time. The diagnoses of AD and aMCI were based on clinical criteria without any pathological or amyloid imaging data. Thus, dementia of other origins may have been included in these diagnoses, particularly aMCI. The large number of excluded subjects due to age-matching and abnormal correlation values between regions is another limitation of this study.

The change of brain network by AD progression and the innate principles are not fully uncovered. The diverse and even opposed results are reported through attempting various methods and measurements. This can be a good time to investigate this issue more seriously to find the causal relationship between brain network and disease progress of AD.

### Conflict of interest statement

The authors declare that the research was conducted in the absence of any commercial or financial relationships that could be construed as a potential conflict of interest.
