# Supplementary material for: Non-monotonic reorganization of brain networks with Alzheimer's disease progression
Source: Front Aging Neurosci. 2015 Jun 9;7:111. doi: 10.3389/fnagi.2015.00111 (PMC4460428; doi:10.3389/fnagi.2015.00111)
Supplement: Supplementary file 1 [file DataSheet1.DOCX]

***Supplementary Material***

**Non-monotonic reorganization of brain networks with Alzheimer’s disease progression**

**HyoungKyu Kim^1§^, Kwangsun Yoo^1§^, Duk L. Na^2, 3^, Sang Won Seo^2, 3^, Jaeseung Jeong^1^*, Yong Jeong^1^***

^1^ Department of Bio and Brain Engineering, Korea Advanced Institute of Science and Technology (KAIST), Daejeon, Republic of Korea

^2^ Department of Neurology, Samsung Medical Center, Sungkyunkwan University School of Medicine, Seoul, Republic of Korea
^3^ Neuroscience Center, Samsung Medical Center, Seoul, Republic of Korea

**§:** These authors contributed equally to this work**.**

*** Correspondence:**

**Yong Jeong, MD, PhD**, Laboratory for Cognitive Neuroscience and NeuroImaging, Department of Bio and Brain Engineering, Korea Advanced Institute of Science and Technology, 291 Daehak-ro, Yuseong-gu, Daejeon, 305-701, Republic of Korea.

yong@kaist.ac.kr

**Jaeseung Jeong, PhD**, Brain Dynamics Laboratory, Department of Bio and Brain Engineering, Korea Advanced Institute of Science and Technology, 291 Daehak-ro, Yuseong-gu, Daejeon, 305-701, Republic of Korea.

jsjeong@kaist.ac.kr

1. **Supplementary Discussion**

## Comment on from the correlation analysis between neuropsychological test scores and network properties

The results from the correlation analysis between neuropsychological test scores and network properties support a stage-specific non-monotonicity. We found that correlations between neuropsychological scores and network properties were distinguishable among each group of AD progression (Table 2, and Supplementary Table S1 and S2). Specifically, the aMCI group showed a significant correlation between attention, language, visuospatial and frontal executive performance and network properties, while other groups exhibited negative correlations only with more narrowly defined clinical criteria (Table 2). Interestingly, only the COWAT semantic score correlated with network topology independent of AD stage (Supplementary Table S2). This finding can be attributed to the widely distributed performance on this measure in the aMCI group compared to the AD group, who showed a floor effect.

## Comment on the reorganization process of the rich-club core with AD progression

We also investigated the reorganization process of the rich-club core with AD progression, which revealed that a prodromal stage of AD, aMCI, distinctively exhibited additional frontal hubs and connections between the frontal and posterior (parietal and occipital) regions. This result would also coincide with the hyperactivation and enhanced functional connectivity in aMCI. Second, we also found that network efficiency was significantly increased from the CDR 0.5 to the CDR 1 stage but decreased again in the subsequent CDR 2 stage (Figure 1). It is interesting but unexpected that network efficiency was reversed at this stage, and the cause of the increase in the CDR 1 group is unclear. This result may indicate that the parameter, network efficiency, is inappropriate for representing the network changes occurring in AD. However, this finding has implications in the context of earlier studies. The results of previous studies investigating the changes in resting connectivity and activation patterns in AD are contradictory; some studies have reported increased hippocampal activation during memory tasks in AD patients compared to HS participants, while other studies have reported the converse ([Pariente et al., 2005](#_ENREF_27);[Johnson et al., 2006](#_ENREF_17)). With respect to resting fMRI studies of hippocampal networks, diverging findings of resting functional connectivity change in AD have also been reported ([Wang et al., 2006](#_ENREF_44);[Zhou et al., 2008](#_ENREF_48);[Bai et al., 2011](#_ENREF_2)). In addition, as mentioned in the introduction, previous graph theoretical analyses have reported inconsistent results across studies (for reviews, ([Xie and He, 2011](#_ENREF_45);[Tijms et al., 2013](#_ENREF_41))). Previous studies have observed decreased, increased, or unchanged clustering coefficients and characteristic path lengths in AD patients. Combined with the enhanced network parameters in the aMCI group, the elevated network efficiency of the CDR 1 group suggests that AD progression occurs in a non-monotonic.

## Comment on other possibilities of non-monotonic changes in AD brain networks

The non-monotonic changes can be explained in other ways. We could assume the progressive loss of synaptic activity, which is represented as a change in edge properties and a progressive loss of neuronal cells, is represented by changes in node properties. With disease progression, the brain is reorganizing the networks with weakened edges and lost nodes to perform functions appropriately, however, at certain stages, this reorganization fails to meet the mission, which leads to progression to the next stage of CDR.

## Comment on the changes in the network properties of each brain lobe

To delineate topological changes, we estimated the changes in the network properties of each brain lobe. This analysis was performed on averaged nodal network properties, and the results indicate that AD progression also induces a non-monotonic change within each lobe or area. As expected, the changes were found mainly in the parietal lobe with respect to efficiency, and in the parietal and frontal lobes in the case of betweenness centrality.

1. **Supplementary Figures and Tables**

## Supplementary Tables

**Supplementary Table 1. The threshold values for the same density network**

| HS | aMCI | AD CDR 0.5 | AD CDR 1 | AD CDR 2 |
| --- | --- | --- | --- | --- |
| 0.831 (±0.042) | 0.833 (±0.042) | 0.841 (±0.037) | 0.840 (±0.072) | 0.849 (±0.044) |

The averaged threshold values for each group and standard deviation.

**Supplementary Table 2. Correlation between network properties and clinical information within each group (p<0.05, p / R^2^ / r)**

|  |  | **Characteristic path length** | **Clustering coefficient** | **Modularity** |
| --- | --- | --- | --- | --- |
| HS | Stroop Test Color-reading Correct | 0.024 / 0.163 / -0.404 | - | - |
| aMCI | Digit Span Backward | 0.028 / 0.097 / -0.311 | - | - |
|  | Naming K-BNT | 0.027 / 0.098 / -0.314 | 0.010 / 0.131 / -0.361 | 0.021 / 0.107 / -0.328 |
|  | RCFT Copy | - | 0.045 / 0.081 / -0.285 | - |
|  | Go/NoGo | 0.028 / 0.097 / -0.311 | 0.009 / 0.133 / -0.365 | - |
|  | COWAT Semantic | 0.009 / 0.134 / -0.366 | 0.005 / 0.155 / -0.393 | 0.012 / 0.124 / -0.352 |
| AD CDR 0.5 | COWAT Phonemic | 0.047 / 0.161 / 0.401 | - | - |

HS: healthy subject / aMCI: amnestic mild cognitive impairment / AD: Alzheimer’s disease / CDR: clinical dementia rating

R^2^: goodness of fit by coefficient of determination / r: Person’s correlation coefficient

**Supplementary Table 3. Correlation between network properties and clinical information without considering groups (p<0.05, p / R^2^ / r))**

|  | **COWAT Semantic** |
| --- | --- |
| Global efficiency | - |
| Local efficiency | - |
| Betweenness centrality | 0.042 / 0.027 / -0.165 |
| Characteristic path length | 0.047 / 0.026 / -0.162 |
| Clustering coefficient | 0.035 / 0.029 / -0.171 |
| Modularity | - |
| Assortativity | - |

R^2^: goodness of fit by coefficient of determination / r: Person’s correlation coefficient

## Suplementary Figures


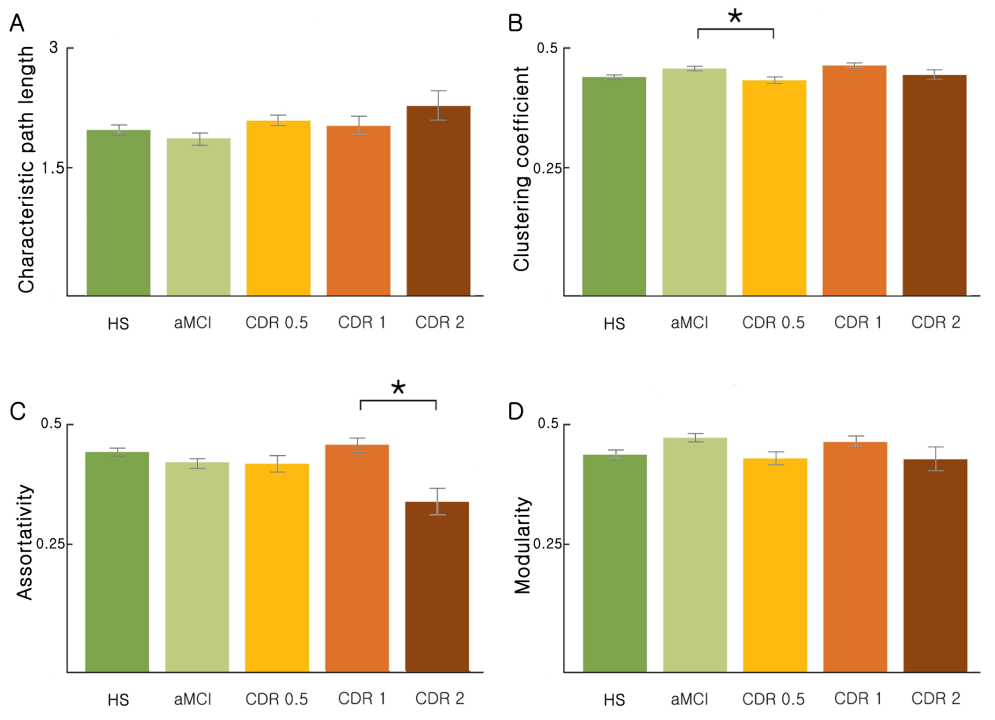


Supplementary Figure 1. Network topological properties with AD progression. (A) Characteristic path length, (B) clustering coefficient, (C) assortativity, and (D) modularity change stage-specifically in a non-monotonic manner. Significance is represented by an asterisk (p<0.05, ANOVA and post-hoc).


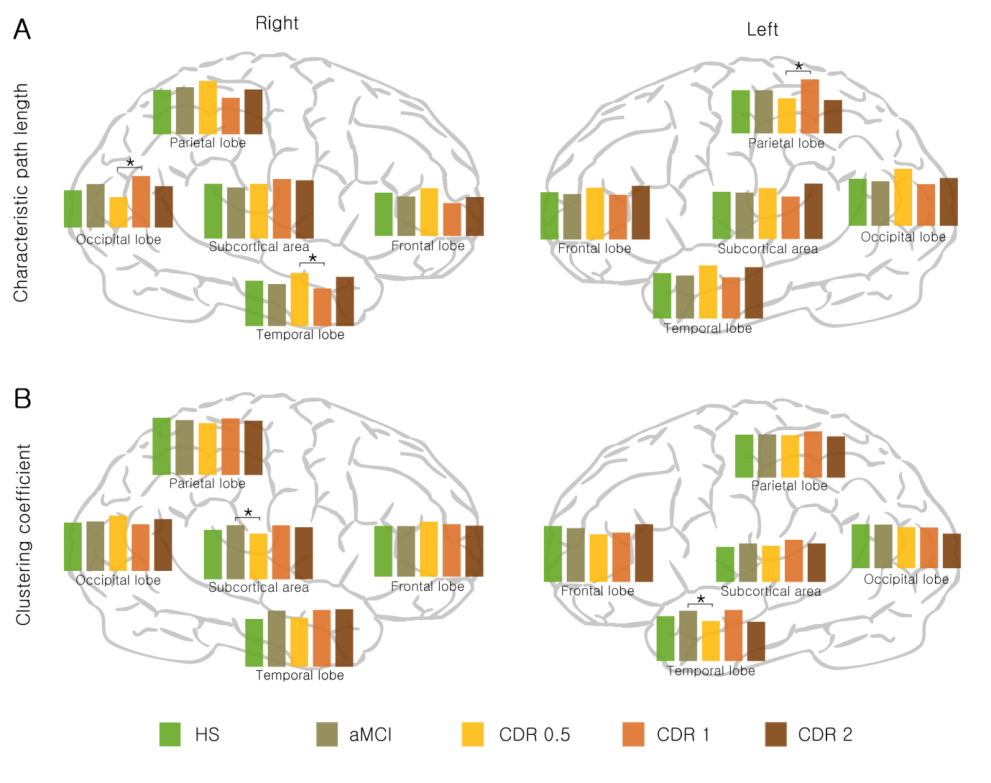


Supplementary Figure 2. Lobar network properties with AD progression. (A) Characteristic path length and (B) clustering coefficient change stage-specifically in a non-monotonic manner. Significance is represented by an asterisk (p<0.05, ANOVA and post-hoc).

1. **References**

Bai, F., Xie, C., Watson, D.R., Shi, Y., Yuan, Y., Wang, Y., Yue, C., Teng, Y., Wu, D., and Zhang, Z. (2011). Aberrant hippocampal subregion networks associated with the classifications of aMCI subjects: a longitudinal resting-state study. *PloS one* 6**,** e29288.

Pariente, J., Cole, S., Henson, R., Clare, L., Kennedy, A., Rossor, M., Cipoloti, L., Puel, M., Demonet, J.F., and Chollet, F. (2005). Alzheimer's patients engage an alternative network during a memory task. *Annals of neurology* 58**,** 870-879.

Johnson, S., Schmitz, T., Moritz, C., Meyerand, M., Rowley, H., Alexander, A., Hansen, K., Gleason, C., Carlsson, C., and Ries, M. (2006). Activation of brain regions vulnerable to Alzheimer's disease: the effect of mild cognitive impairment. *Neurobiology of aging* 27**,** 1604-1612.

Tijms, B.M., Wink, A.M., De Haan, W., Van Der Flier, W.M., Stam, C.J., Scheltens, P., and Barkhof, F. (2013). Alzheimer's disease: connecting findings from graph theoretical studies of brain networks. *Neurobiology of aging* 34**,** 2023-2036.

Wang, L., Zang, Y., He, Y., Liang, M., Zhang, X., Tian, L., Wu, T., Jiang, T., and Li, K. (2006). Changes in hippocampal connectivity in the early stages of Alzheimer's disease: evidence from resting state fMRI. *Neuroimage* 31**,** 496-504.

Xie, T., and He, Y. (2011). Mapping the Alzheimer's brain with connectomics. *Front Psychiatry* 2**,** 77. doi: 10.3389/fpsyt.2011.00077.

Zhou, Y., Dougherty Jr, J.H., Hubner, K.F., Bai, B., Cannon, R.L., and Hutson, R.K. (2008). Abnormal connectivity in the posterior cingulate and hippocampus in early Alzheimer's disease and mild cognitive impairment. *Alzheimer's & Dementia* 4**,** 265-270.
